# Genetic enhancement of behavioral itch responses in mice lacking phosphoinositide 3-kinase-γ (PI3Kγ)

**DOI:** 10.1186/1744-8069-7-96

**Published:** 2011-12-14

**Authors:** Bolam Lee, Giannina Descalzi, Jinhee Baek, Jae-Ick Kim, Hye-Ryeon Lee, Kyungmin Lee, Bong-Kiun Kaang, Min Zhuo

**Affiliations:** 1 Department of Brain and Cognitive Sciences, Seoul National University, Seoul 151-747, Korea; 2 National Creative Research Initiative Center for Memory, Department of Biological Sciences, College of Natural Sciences, Seoul National University, Seoul, Korea; 3 Department of Physiology, Faculty of Medicine, University of Toronto Centre for the Study of Pain, 1 King's College Circle, Toronto, ON, Canada; 4 Department of Anatomy, School of Medicine, Kyungpook National University, 2-101 Dongin-Dong, Daegu, Korea

## Abstract

Phosphoinositide 3-kinases (PI3Ks) are important for synaptic plasticity and various brain functions. The only class IB isoform of PI3K, PI3Kγ, has received the most attention due to its unique roles in synaptic plasticity and cognition. However, the potential role of PI3Kγ in sensory transmission, such as pain and itch has not been examined. In this study, we present the evidence for the first time, that genetic deletion of PI3Kγ enhanced scratching behaviours in histamine-dependent and protease-activated receptor 2 (PAR-2)-dependent itch. In contrast, PI3Kγ-deficient mice did not exhibit enhanced scratching in chloroquine-induced itch, suggesting that PI3Kγ selectively contributes to certain types of behavioal itch response. Furthermore, PI3Kγ-deficient mice exhibited normal acute nociceptive responses to thermal and mechanical noxious stimuli. Behavioral licking responses to intraplantar injections of formalin and mechanical allodynia in a chronic inflammatory pain model (CFA) were also not affected by PI3Kγ gene deletion. Our findings indicate that PI3Kγ selectively contributes to behavioral itching induced by histamine and PAR-2 agonist, but not chloroquine agonist.

## Introduction

PI3Ks are lipid kinases that phosphorylate the 3'-hydroxyl group of the inositol rings in phophatidylinositol (PtdIns) substrates [1], and they also possess protein kinase activity. PI3K signaling is well known for its multiple functions, including vesicle trafficking, cell metabolism, and cell growth and survival [2]. PI3Ks are classified as class I, class II, or class III based on substrate binding and sequence homology; class I PI3Ks are subdivided into α, β, γ, and δ [2]. PI3Ks work as important intracellular messengers, and may act downstream of signaling proteins involved in the expression of long term potentiation (LTP), or long term depression (LTD) [3,4]. For example, PI3K antagonists block LTP expression in the CA1 region of the hippocampus [5]. More recently, PI3K activity was shown to mediate stress-induced impairments in hippocampal LTP and to facilitate stress-induced potentiation of hippocampal LTD in adult rats [3]. Furthermore, previous observations revealed that PI3K blockade prevents both AMPA and NMDA receptor-mediated ERK1/2 activation [6]. Interestingly, a recent genetic and pharmacological study showed that PI3Kγ is specifically involved in NMDA receptor-dependent LTD and behavioural flexibility in the hippocampus [7].

In addition to the hippocampus, PI3K has also been reported in brain areas related to sensory transmission and modulation. For example, PI3Kγ has been found to be expressed in cortical areas including the anterior cingulate cortex (ACC), insular cortex and somatosensory cortex [8]. In addition, various studies have identified PI3K activity within the spinal cord [9,10]. Specifically, PI3Kγ has been detected in a subtype of C-fibers in dorsal root ganglion (DRG) neurons [11], and PI3Kγ expression in the dorsal horn of the spinal cord has been reported (The Allen Mouse Brain Atlas). Therefore, PI3Kγ likely localizes with somatosensory pathways and may contribute to sensory transmission, modulation and plasticity. In accordance, activation of PI3K has been reported in nociception [9,10,12]. Previous studies suggest that PI3K may activate downstream of key signaling proteins involved in nociceptive sensitization, such as nerve growth factor (NGF). For example, NGF mediated TRPV1 receptor sensitization to capsaicin has been found to be absent in mice lacking the PI3K regulatory subunit p85α [9]. Similarly, pharmacological interventions have also revealed that PI3K inhibition can attenuate GDNF mediated hyperalgesia [12]. Furthermore, PI3K activity was found to be significantly increased in DRG neurons after spinal nerve injury, and selective PI3K inhibition attenuated behavioral manifestations of chronic pain [10]. Nevertheless, most of these observations have employed pharmacological inhibitors that are not selective for specific subtypes of PI3K. Therefore, possible somatosensory contributions of selective subtypes, such as PI3Kγ have yet to be investigated.

Itch is also a complex, unpleasant sensory experience that plays an important role in protecting organisms against potentially harmful agents [13]. Increasingly, research into itch has begun to uncover its molecular mechanisms, and it has become apparent that distinct molecular substrates may exist for itch or pain, but share some common signalling pathways (Table 1). For example, superficial dorsal horn neurons in rats were found to respond to both histamine and capsaicin stimulation [14], (but see ref [15] for cat). Similarly, peripheral serotonin stimulation has been shown to elicit scratching behaviour and *c-fos *in lamina I-III neurons of adult rats [16,17]. Correspondingly, several studies have observed that histamine and other puritogens activate superficial dorsal horn neurons in adult mice [13,18,19]. The gastrin releasing peptide (GRP) receptor (GRPR) has emerged as a possible itch specific mediator at the spinal cord [20]. Gene knockout mice lacking GRPRs, display a significant reduction in scratching behavior in response to peripheral application of histamine and other pruritogens compared to wild type littermates, but retain intact mechanical and thermal nociception. However, our recent studies found that glutamate, a key excitatory transmitter for sensory nociceptive transmission, is also a key neurotransmitter for GRP-dependent and -independent synaptic transmission in the mammalian spinal cord [19]. Thus, it is likely that at the spinal cord level, both pain and itch employ glutamate as a common fast excitatory transmitter. It is thus unlikely that there are selectively labeled sensory pathways or transmitter for itch or pain.

**Table 1:** Recent itch studies using genetically engineered mice

Deleted gene	Pruritogens	Scratching	Reference
**PLCβ3**	Histamine, HTMT, Compound 48/80, Clobenpropit	Reduced	Neuron (2006)
**GRPR**	Compound 48/80, PAR-2, chloroquine	Reduced	Nature (2007)
**VGLUT2** **(Spinal cord specific)**	PAR-2, 5-HT, Chloroquine, Compound 48/80, Capsaicin	Increased	Neuron (2010)
**Bhlhb5**	Histamine, Compound 48/80 PAR-2, 5-HT, Chloroquine	Increased	Neuron (2010)
**TLR7**	5-HT, ET-1, PAR-2, Chloroquine	Reduced	Nature Neuroscience (2010)
**TRPA1**	Chloroquine, BAM8-22	Reduced	Nature Neuroscience (2011)
**PI3Kγ**	istamine, PAR-2	Increased	The present study

Considering the role of PI3K and PI3Kγ in synaptic plasticity of glutamatergic synapses, we wanted to test if subtypes of PI3K may preferentially contribute to itch. In the present study, we investigated the behaviors of PI3Kγ knock-out mice to characterize the role of PI3Kγ in itch responses. We studied the effects of several pruritogens on PI3Kγ knockout mice and identified several itch-specific phenotypes with relatively intact pain responses. We discovered that PI3Kγ is an essential molecule for histamine-dependent and protease-activated receptor 2 (PAR-2)-dependent itch behaviors, but is not essential to chloroquine-dependent itch behaviors.

## Methods

### Animals

Male and female PI3Kγ KO and WT littermates with a C57BL/6J genetic background (back-crossings were executed over 12 generations) were used for behavioral experiments. We received PI3Kγ mice initially from Peter Backx, and backcrossed them to C57BL/6J more than 15 times in our mouse facilities. All mice were adults (8-12 weeks old) and housed under a 12:12 light cycle with food and water provided *ad libitum*. The Animal Care and Use Committee of Seoul National University approved all mouse protocols.

### Nociceptive behavioral tests

All behavior experiments were performed under blind conditions. The hot plate test was performed by placing a mouse on a 48°C or 53°C hot plate (Harvard apparatus) and measuring the latency of hind limb response, such as flinching, licking, and jumping. The maximum time allowed at 53°C was 30 seconds to prevent tissue damage; if the animal did not respond within this time, the animal was excluded.

Mechanical threshold was tested under non-restrained conditions. Mice were allowed to acclimate to the elevated wire mesh for 15 minutes before testing. A threshold stimulus was determined by observing animal hind paw withdrawal upon application of a von Frey filament (Touch-Test kit, North Carolina, Inc.), and the threshold was calculated with "up-and-down paradigm" [21]. Positive responses included prolonged hind paw withdrawal and licking or biting of the hind paw. Mechanical allodynia was tested on days 1, 3, and 7 after injection of Complete Freund's adjuvant (CFA, 10 μl, Sigma) into the plantar of the right hind paw.

Formalin (5%, 10 μl) was injected as same as CFA. The total time spent licking, flinching, and shaking the injected area was recorded for each 5-minute interval over 2 hours.

### Itch behavioral tests

Itch behavior was recorded with a digital video camera for 3-40 minutes. Each mouse was used only once, in one experiment. All animals were habituated to the observation chamber (200 × 260 × 130 mm) with proper bedding for 30 minutes before testing. Pruritogens, histamine (250 μg), PAR-2 agonist, SLIGRL-NH_2 _(100 μg), and chloroquine (200 μg) were used. Each chemical was injected intradermally into the nape of the neck via a 30-gauge Hamilton syringe in a volume of 20-25 μl. One bout of scratching by either hind paw was defined as a scratching episode. The scratching was quantified as the total number of scratches plus the cumulative number of scratches during 5-minute intervals over 3-40 minute observation periods.

### Pharmacological tests

The PI3Kγ specific inhibitor, AS605240 (Calbiochem), was dissolved in DMSO (Sigma) and the drug concentration was adjusted with PBS. The total volume for intraperitoneal injection was 200 μl from a 0.3-ml insulin syringe (31-gauge). The inhibitor or vehicle was injected intraperitoneally 30 minutes before behavioral experiments, and the mice were allowed to adapt to the testing cage immediately following injection.

### Data Analysis

Results are presented as the mean ± standard error of the mean (s.e.m.). Statistical comparisons were performed by one- or two-way analysis of variance (ANOVA). For the total number of itch behaviours and basal mechanical thresholds, unpaired Student's *t*-test was used. In all cases p < 0.05 was considered statistically significant.

## Results

### Acute nociception

**Figure 1:**
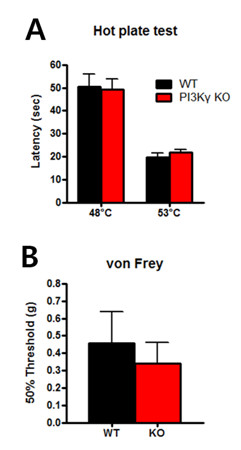
**Intact acute nociception in PI3Kγ-deficient mice**. (A) Hot plate test results show that PI3Kγ KO and WT littermates have similar behavioral responses to thermal stimuli. (B) Basal mechanical threshold was measured and no significant differences were detected between the groups.

To investigate PI3Kγ involvement in acute nociception, we measured basal thermal and mechanical sensitivity in PI3Kγ knockout (KO) mice and wild-type (WT) littermates. The hot-plate (HP) test showed similar paw withdrawal latencies at 48°C and 53°C in both groups (HP at 48°C, WT = 50.6 ± 5.2 sec, KO = 49.2 ± 4.7 sec; HP at 53°C, WT = 19.6 ± 2.0 sec, KO = 21.6 ± 1.4 sec; WT n = 10, KO n = 7; two-way ANOVA, genotype factor, *p *> 0.05, Figure 1A). Next, we measured behavioural responses to mechanical stimuli. No significant differences in the withdrawal response were observed between PI3Kγ KO and WT mice (WT = 0.46 ± 0.18 g, KO = 0.34 ± 0.12 g; n = 7 for each group; unpaired *t*-test, *p *> 0.05, Figure 1B). Taken together, these results suggest that PI3Kγ does not contribute to acute nociception in mice.

### Normal range of nociception to chemical inflammation

**Figure 2:**
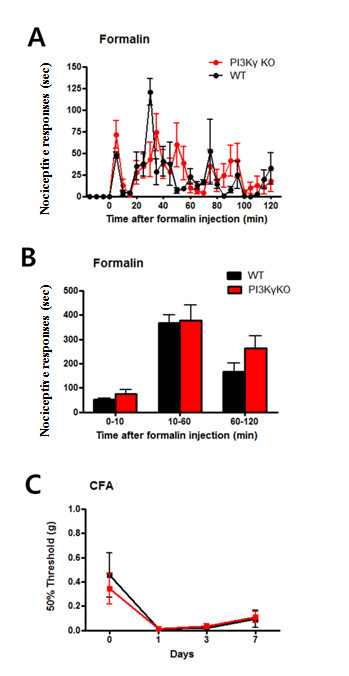
**Intact chemical-induced pain in PI3Kγ-deficient mice**. (A and B) Formalin-induced inflammatory pain response indicates that PI3Kγ KO and WT littermates have no significant nociceptive differences. (C) CFA-induced allodynia was measured 1, 3, and 7 days after the injection, and no significant differences were observed in hypersensitivity to mechanical touch.

Considering the important roles of PI3K on synaptic plasticity, we then examined the responses of PI3Kγ KO and WT to more prolonged nociceptive stimulus, peripheral formalin injection. Formalin is a common model for tissue injury and inflammation [21]. To test formalin-induced pain, intraplantar injection (5%, 10 μl) was made in the right side of the hind paw. As we reported before [21], mice responded by licking the injected hindpaw, and this behaviour is typically concentrated in three distinct phases: a first phase (0-10 min), a second phase (10-55 min) and a third phase (55-120 min). We thus recorded their pain response for 2 hours after formalin injection to observe any long-term effects of sensitization. We found that there were no significant differences in response to formalin between the groups (WT n = 6, KO n = 9; two-way repeated measures ANOVA, genotype factor, *p *> 0.05, Figure 2A). Interestingly, the formalin graph shows a slight trend towards an increased pain response in PI3Kγ KO mice during the phase 3, but the mean difference was not statistically significant (WT = 165.9 ± 35.6 sec, KO = 262.3 ± 51.9 sec; unpaired *t*-test, *p > 0.05*, Figure 2A-B).

Next, CFA was used to study chronic inflammatory pain behaviour as we have previously examined. Behavioural allodynia was measured 1, 3, and 7 days after the intraplantar CFA injection. Both PI3Kγ KO and their WT littermates displayed similar levels of hyper mechanical sensitivity to a series of von Frey filaments. No significant differences were observed in the reduced mechanical threshold (n = 7 for each group; two-way ANOVA, genotype factor, *p *> 0.05, Figure 2C). These results suggest that PI3Kγ activity is not required for behaviroal responses to inflammatory injury.

### Abnormal scratching behaviour induced by histamine and PAR-2 agonist

**Figure 3:**
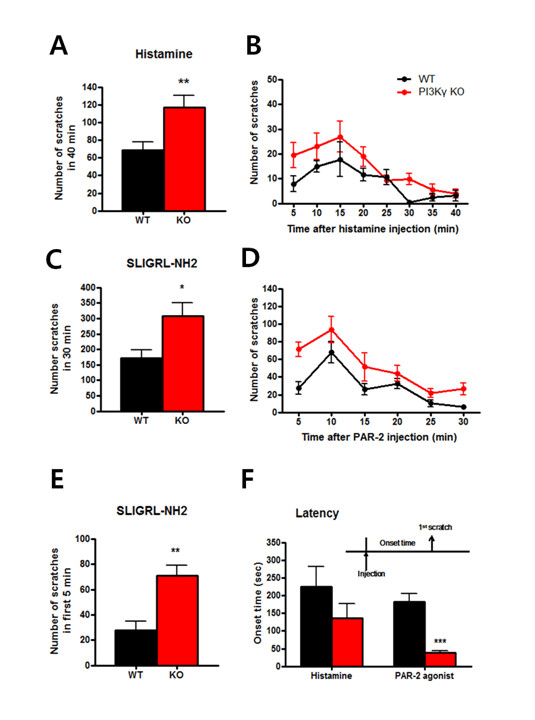
**Histamine and PAR-2-induced hyper scratching in PI3Kγ deficient mice**. (A and B) The total number of scratches that occurred over a 40-minute time period after histamine injection were counted. PI3Kγ KO exhibited enhanced scratching compared to WT littermates. (C and D) PAR-2 agonist induced itch was recorded; scratches were counted for 30 minutes. (E and F) The PI3Kγ KO scratched vigorously for the first 5 minutes; the latency until the first scratch was significantly earlier than in WT mice.

Histamine is well-known pruritogen that induces scratching behaviour, and it has been used to identify itch receptors in the human skin [22]. Recently, rodent model has been developed to satisfy clinical needs in the field of itch and enabled genetic studies [23]. To examine if the loss of PI3Kγ affects behavioural itch, we conducted initial experiments with histamine. Scratching behaviour was recorded for 40 minutes, and the number of scratches was counted in a blind manner. We observed itch behaviour for 40 minutes because most of the mice barely scratched after then. As previously reported [24], all mice started scratching behaviour after the intradermal injection of histamine. Surprisingly, the scratching in PI3Kγ KO mice was significantly enhanced overall. As a result, the total number of scratches for 40 minutes was significantly higher in the PI3Kγ KO group compared to their WT littermates (total number of scratches, WT = 68.8 ± 9.5, KO = 117.2 ± 13.3; n = 10 for each group; unpaired *t*-test, *p *< 0.01; number of scratches, two-way repeated measures ANOVA, genotype factor, *p *< 0.001, Figure 3A-B).

Other well-known pruritogen proteases were chosen to study if histamine-independent signaling is also related to PI3Kγ deficiency. A recent study showed that agonists of protease-activated receptor 2 (PAR2) induce itch [25]. We performed another set of itch experiments with the PAR-2 agonist, SLIGRL-NH2 and observed scratching behavior for 30 minutes. In consistent with the previous results, both WT and KO mice exhibited increased scratching behavior triggered by the PAR-2 agonist injection. Interestingly, PI3Kγ KO mice exhibited vigorous scratches compared to the WT group (total number of scratches, WT = 171.2 ± 26.2, KO = 308.7 ± 41.3; unpaired *t*-test, *p <*0.05; number of scratches, two-way repeated measures ANOVA, genotype factor, *p *< 0.05, Figure 3C-D). PI3Kγ KO mice particularly displayed hyper scratching during the first 5 minutes, and the mean difference between the groups during the first 5 minutes was significantly different (scratches during the first 5 minutes, WT = 27.7 ± 7.1, KO = 70.9 ± 8.1; unpaired *t*-test, *p *< 0.01, Figure 3E). Additionally, PI3Kγ KO mice displayed significantly shorter scratching latencies than WT littermates, which were followed by consistent, vigorous scratching behaviour throughout the experiment (WT = 185.8 ± 24.4, KO = 39.1 ± 4.9; unpaired *t*-test, *p *< 0.001, Figure 3F). The reason that this early onset of scratch in PAR-2 dependent itch is interesting is because no differences were detected in scratching latencies in response to the histamine application. Therefore, PAR-2 dependent itch implies a unique mechanism with PI3Kγ and suggests a pivotal role of PI3Kγ in protease-mediated itch.

### Normal scratching in chloroquine-induced itch

**Figure 4:**
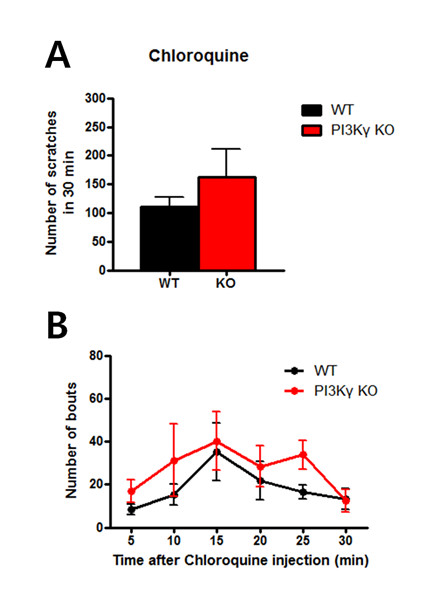
**Similar scratch levels in chloroquine-induced itch**. (A) No significant differences were observed in the number of scratches found in chloroquine-induced itch experiments with PI3Kγ KO and WT littermates. (B) Enhanced itch behaviors following chloroquine injection were not observed during the 30-minute time period following injection.

Chloroquine is a widely used antimalarial medication, but it has a serious side effect that patients often have itch. A recent study investigated chloroquine-mediated itch pathway in mice [26], and chloroquine was found to bind sensory neuron-specific GPCR Mrgprs. In addition, another study showed that TRPA1 is required to itch response induced by chloroquine [27]. To characterize PI3Kγ in the function of itch, we used chloroquine as another type of histamine-independent itch model. Chloroquine was injected intradermally and subsequent behavioral scratching responses were recorded in the same fashion as the above experiments. Unlike previous results with histamine and PAR-2 agonism, chloroquine-induced itch did not trigger hyper scratching behavior in PI3Kγ KO mice compared to WT littermates (WT = 110.3 ± 17.0, KO = 163.1 ± 48.5; unpaired *t*-test, *p *> 0.05, Figure 4A; two-way repeated measures ANOVA, genotype factor, *p *> 0.05, Figure 4B). This negative result implies that the chloroquine receptors may reside in the sensory transmission pathway irrespective of PI3Kγ. Overall, the absence of PI3Kγ did not affect chloroquine-induced scratching.

### Pharmacological inhibition of PI3Kγ

**Figure 5:**
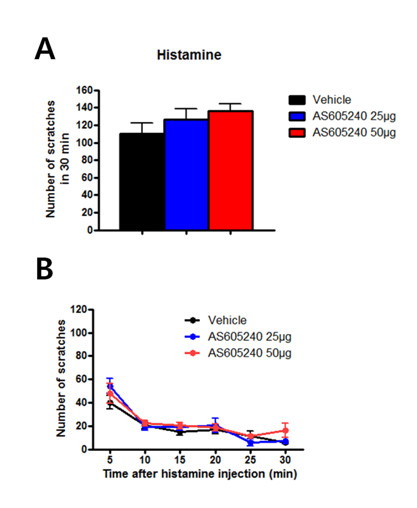
**Acute pharmacological inhibition of PI3Kγ and histamine-dependent itch test**. (A-B) Intraperitoneal injection of PI3Kγ selective inhibitor, AS605240, prior to an itch experiment did not induce significantly enhanced scratches in the drug group compared to the vehicle group.

To investigate if acute pharmacological inhibition of PI3Kγ in naïve mice results in enhanced scratching, we treated naïve mice with the PI3Kγ-specific inhibitor, AS605240 [28], and conducted an itch experiment with histamine. Adult naïve mice were injected intraperitoneally with either inhibitor or vehicle. The pharmacological inhibition of PI3Kγ prior to the itch test did not lead to significantly enhanced scratches (n = 12 for each group; one-way ANOVA, *p >*0.05, Figure 5A-B). Since the experiments for the higher dose group (50 μg) and the vehicle group were conducted simultaneously, we were able to analyze the difference in the number of scratches between the two groups. The number of scratches appeared to be higher in the higher dose group (50 μg) compared to the vehicle group, but the numbers did not reach statistical significance (vehicle = 110.7 ± 11.9, 50 μg of drug = 136.6 ± 8.2; unpaired *t*-test, *p = *0.08, Figure 5A). One possible difference between results of PI3Kγ KO mice and the PI3Kγ inhibitor is that pharmacological inhibition is not complete or specific. Future studies are clearly needed when more selective inhibitors become available.

## Discussion

This is the first study identifying PI3Kγ as a mediator of itch sensation in the mammalian system. We report that PI3Kγ-deficient mice exhibit enhanced scratching in response to intradermal injections of histamine and PAR-2 agonists compared to WT littermates. Furthermore, PI3Kγ-deficient mice demonstrated significantly shorter latencies for scratching and much more pronounced scratching behaviors in response to PAR-2 agonist treatment. In contrast, acute mechanical and thermal thresholds were unaltered by PI3Kγ deletion, and chemical-induced pain responses resembled that of WT littermates. Surprisingly, enhanced scratching was not observed in PI3Kγ KO mice during the chloroquine-induced itch experiments, suggesting a distinct intracellular mechanism for this form of itch behavior.

### Spinal PI3K, pain and itch

Pain and itch mechanisms have long been suspected to overlap. Mounting evidence suggests that C-fibre innervation provides pruritogenic transmission, and we have previously shown that GRP sensitive neurons almost exclusively receive primary afferent C-fiber input; whereas GRP insensitive neurons mostly received A_δ _fiber input. However, electrophysiological studies revealed that sensory transmission in both GRP-sensitive or insensitive neurons receive glutamatergic innervations. These findings suggest that GRP is not a selective transmitter for itch as originally proposed [20]. This notion is further supported by the observation that itch and pain transmission can be modulated through TRPV1-expressing primary afferents [22]. Histamine induced activation was shown to be TRPV1 dependent in mouse primary afferent neurons [23]. In accordance, NGF mediated TRPV1 channel sensitization to capsaicin is sensitive to disruptions in PI3K levels [9]. Similarly, a histamine-independent pruritogen, protease-activated receptor 2 agonist (PAR-2), is another well-known itch inducer and has been reported to sensitize TRPV1 channels to induce hyperalgesia in small-to-medium diameter neurons of the DRG [24]. Thus it is possible that PI3Kγ may contribute to itch-related sensitization at peripheral sites.

Much research has presented evidence of central sensitization in chronic pain [25,26] and chronic itch [13]. Chronic pain has been found to manifest through sustained potentiation of nociceptive systems. PI3K activity was found to significantly increase in DRG neurons in response to spinal nerve injury, and selective PI3K inhibition successfully attenuated behavioral manifestations of chronic pain [10]. Therefore, it is possible that PI3Kγ may mediate its effects by affecting glutamate release mechanisms. Indeed, several lines of evidence suggest a role for glutamate transmission in itch sensation [12,19]. VGLUT2 deficient mice show robust itch phenotypes, whereby scratching behaviour becomes so pronounced that tissue damage develops [10]. More recently, we showed that C-fiber evoked responses in GRP sensitive neurons within the dorsal horn were sensitive to CNQX [19]. In contrast, VGLUT2-dependent glutamate release from DRG neurons has been reported to suppress itch [27].

### Cortical and subcortical contributions

PI3Kγ is expressed in the cortex [8], and has been implicated in synaptic plasticity related events within the hippocampus. The robust potentiation of itch behaviour in PI3Kγ KO mice could be explained through a reduction of excitatory input to inhibitory neurons. In the ACC, GRP has been shown to increase the frequency of sIPSCs [28], and it would be of interest to see if PI3Kγ mediates this facilitation. The implication that PI3Kγ may mediate glutamatergic transmission corresponds well with previous observations that PI3K activity is critical for AMPA receptor insertion in hippocampal LTP [29]. More recently, it was observed that PI3K inhibition attenuates Ca^2+^-permeable AMPA receptor mediated LTP at CA1 hippocampal synapses in adult mice [30]. Furthermore, stress was observed to induce hippocampal PI3K activity that corresponded with both an attenuation of LTP and facilitation of LTD, whereas intra-hippocampal injections of the PI3K inhibitor LY294002 blocked these effects [3]. Therefore, PI3Kγ could mediate pruritogenic mechanisms through the regulation of inhibitory neuronal activity, whereby a reduction of PI3Kγ prevents AMPA receptor driven GABAergic neuronal activity.

### Functional implications

Chronic pruritus is a debilitating condition that is poorly understood. Recent reports suggest that the *N*-methyl-D-aspartate (NMDA) glutamate receptor may be a therapeutic target for the treatment of itch, but although effective, it is greatly hampered by the numerous undesirable side effects to the central nervous system [31]. Thus P13Kγ may present a novel pharmacological target to modulate itch specific responses through targeting pruritogenic receptor mediated alterations in glutamatergic transmission.

## List of Abbreviations

PI3K: phosphoinositide 3-kinases; DRG: dorsal root ganglion; PAG: periaqueductal gray matter; GRP: gastrin releasing peptide; GRPRs: gastrin releasing peptide receptors; TRPV1: transient receptor potential vanilloid 1; VGLUT2: vesicular glutamate transporter subtype 2; PAR-2: protease-activated receptor 2; GPCR: G-protein coupled receptor; NGF: nerve growth factor

## Competing interests

The authors declare that they have no competing interests.

## Authors' contributions

BL performed most of the behavioural experiments and wrote the manuscript and JB and JI took charge of PI3Kγ mice breeding, genotyping and housing condition. HR supervised BL mouse handling skills and result discussion. GD also set up behavioral protocols and wrote the manuscript. KM, BK, and MZ organized research design and edited the final draft of the manuscript. All authors have read and approved the final manuscript.
